# Use of Number by Fish

**DOI:** 10.1371/journal.pone.0004786

**Published:** 2009-03-10

**Authors:** Christian Agrillo, Marco Dadda, Giovanna Serena, Angelo Bisazza

**Affiliations:** Department of General Psychology, University of Padova, Padova, Italy; L'université Pierre et Marie Curie, France

## Abstract

**Background:**

Research on human infants, mammals, birds and fish has demonstrated that rudimentary numerical abilities pre-date the evolution of human language. Yet there is controversy as to whether animals represent numbers mentally or rather base their judgments on non-numerical perceptual variables that co-vary with numerosity. To date, mental representation of number has been convincingly documented only for a few mammals.

**Methodology/Principal Findings:**

Here we used a training procedure to investigate whether mosquitofish could learn to discriminate between two and three objects even when denied access to non-numerical information. In the first experiment, fish were trained to discriminate between two sets of geometric figures. These varied in shape, size, brightness and distance, but no control for non-numerical variables was made. Subjects were then re-tested while controlling for one non-numerical variable at a time. Total luminance of the stimuli and the sum of perimeter of figures appeared irrelevant, but performance dropped to chance level when stimuli were matched for the cumulative surface area or for the overall space occupied by the arrays, indicating that these latter cues had been spontaneously used by the fish during the learning process. In a second experiment, where the task consisted of discriminating 2 vs 3 elements with all non-numerical variables simultaneously controlled for, all subjects proved able to learn the discrimination, and interestingly they did not make more errors than the fish in Experiment 1 that could access non-numerical information in order to accomplish the task.

**Conclusions/Significance:**

Mosquitofish can learn to discriminate small quantities, even when non-numerical indicators of quantity are unavailable, hence providing the first evidence that fish, like primates, can use numbers. As in humans and non-human primates, genuine counting appears to be a ‘last resort’ strategy in fish, when no other perceptual mechanism may suggest the quantity of the elements. However, our data suggest that, at least in fish, the priority of perceptual over numerical information is not related to a greater cognitive load imposed by direct numerical computation.

## Introduction

Abilities such as recording the number of events, enumerating items in a set, or comparing two different sets of objects, can be adaptive in a number of ecological contexts. Lyon [1], for instance, reported a spontaneous use of numerical information (egg recognition and counting) in a natural context as a strategy to reduce the costs of conspecific brood parasitism in American coots.

McComb and co-workers [2] using playback experiments found that wild lions based the decision whether or not to attack a group of intruders on a comparison of the number of roaring intruders they had heared and the number and composition of their own group. Over the last two decades or so, extensive laboratory research carried out on monkeys and apes [3]–[6] has revealed the existence of non-verbal systems of numerical representation that non-human primates apparently share both with human infants and with human adults tested in comparable conditions [7], [8]. In recent years, rudimentary numerical abilities have been reported in several other mammalian and avian species, among others, elephants, dolphins, dogs, cats, robins and chicks [9]–[14].

Recently we found [15] that fish, seeking safety from predators, display a rudimentary numerical ability in selecting the largest shoal. Interestingly, the limits shown by fish in this task closely resemble those that have been reported for primates. These data in particular suggest the existence, as in primates, of two independent pre-verbal systems: one for counting a small quantity (≤4) precisely, and the other for estimating large quantities (>4) approximately. These findings suggest the possibility that all extant vertebrates share similar quantificational mechanisms, which may have an ancient phylogenetic origin, at least predating the divergence of the tetrapod lineage.

Nonetheless, before concluding that the same systems are involved, it is necessary to understand whether similar limits really reflect identical underlying mechanisms. In particular, it has been extensively demonstrated that both humans and nonhuman animals can discriminate between two quantities without necessarily counting the number of objects. Numerosity normally co-varies with several other physical attributes, and organisms can use the relative magnitude of continuous variables such as the total area of the stimuli or the sum of their contour, to estimate which group is larger/smaller [16]–[18]. Discriminations based on number or on continuous extent often yield comparable results and therefore carefully controlled experiments are necessary to show that an animal is really using numerical information. Experiments of this type demonstrate that, when selecting the larger shoal, mosquitofish (*Gambusia holbrooki*) spontaneously use non-numerical cues, namely the sum of areas of the shoals and the overall quantity of movements of the individuals within the shoal [15]. This does not necessarily imply that fish are unable to discriminate two groups on the basis of the numerosity alone. Overall perceptual cues may simply be the easiest indicators of numerosity in this task. Indeed, there is persuasive evidence that even humans and non-human primates, which have the capacity to represent number, in many circumstances base their quantity judgment primarily on proxy measures such as area, contour or density of elements and that they use number as a last resort, when there are no other available cues [19], [20].

In the present work we investigated whether fish can discriminate between two quantities when access to non-numerical cues was prevented. The procedure used in our previous studies with fish did not easily permit a fine-grained manipulation of stimuli and an efficient control of continuous perceptual variables that correlate with number. Therefore we adopted a procedure modelled on carefully-controlled experiments conducted on non-human primates, which consisted of training the subject to discriminate between sets containing different numbers of geometric figures while controlling for the perceptual non-numerical variables [3], [21].

The first experiment aimed to determine which cues mosquitofish used spontaneously when both numerical information and continuous physical attributes are available. Subjects learned a discrimination between 2 and 3 objects in the absence of any manipulation of the stimuli; after animals had achieved learning criterion they were tested without reward while controlling for one perceptual non-numerical variable at a time. In the second experiment we trained fish to discriminate between 2 and 3 objects while we simultaneously controlled for non-numerical variables, in order to determine whether fish could discriminate quantities by using only numerical information as shown for mammals.

## Results

### Experiment 1.a. Cues spontaneously used by fish to discriminate between quantities

**Figure 1:**
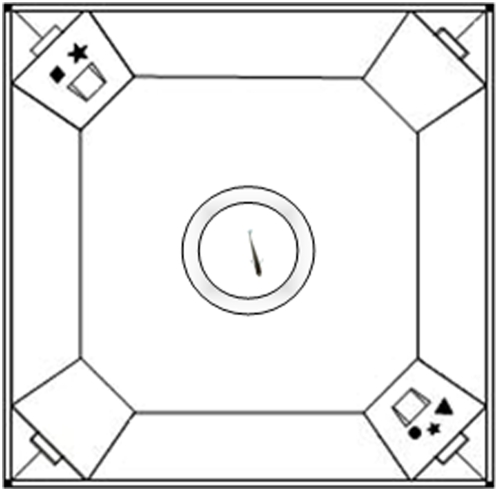
Apparatus used to train fish. Subjects were singly placed in the middle of a test chamber provided with two doors (one associated to three and the other associated to two elements) placed at two opposite corners. Subject could pass through the reinforced door to rejoin shoal mates in the outer tank (not shown).

**Figure 2:**
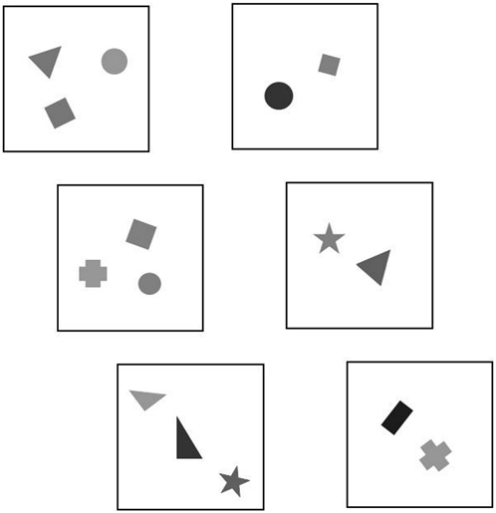
Schematic representation of the stimuli used in experiment 1. Each pair was composed of one set of two and one of three elements. Elements varied in shape, size, brightness and position, and were randomly selected from a large pool.

Ten female mosquitofish were placed in an unfamiliar tank and trained to discriminate between two doors in order to re-join their social group (Fig. 1). Doors were associated with a pair of stimuli consisting of two or three small figures (Fig. 2). These figures were randomly selected with replacement from a pool of approximately 100, and no control for non-numerical variables was operated in the learning phase. Subjects were given six trials per day for a maximum of ten days. Once a subject had reached the learning criterion, it was admitted to the test phase and was examined in the same apparatus without reward (no possibity to re-join the conspecifics) while controlling for one perceptual non-numerical variable at time. We controlled those variables that were shown to be relevant in previous studies with vertebrates, namely the total luminance of the two stimuli, the sum of perimeter of the figures, the cumulative surface area, and the overall space occupied by the arrays. Since operant conditioning is normally a stressful procedure for fish, we adopted a pre-training procedure that consisted of exposing the subjects, in the seven days preceding the training, to the choice of similar pairs of stimuli in order to move from one compartment to the other of their home-tank.

**Figure 3:**
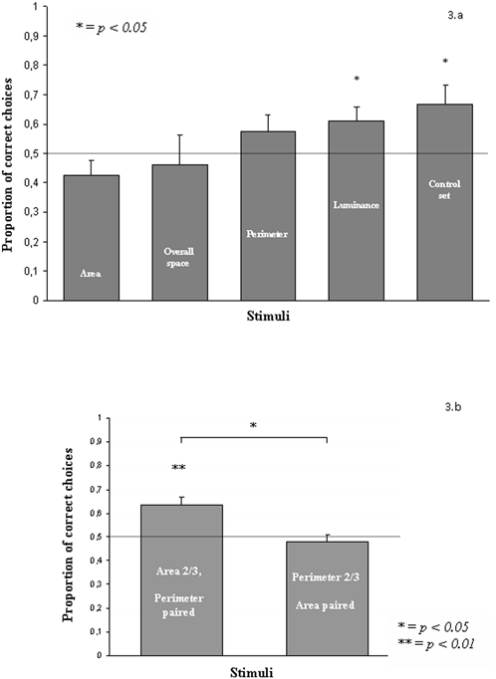
Results of Experiment 1. (a) Proportion of correct choices when area, space, perimeter and luminance were singly controlled for (exp 1 a). (b) Proportion of correct choices when area and perimeter were controlled for (exp 1b).

All ten subjects reached the learning criterion in the training phase, but one was excluded from the subsequent test phase due to poor health, and hence nine started the test phase. We reported no difference in the proportion of correct choices between fish trained with three (mean±std. dev.: 0.753±0.065) and those trained with two figures (0.678±0.028; t(7) = 2.337, p = 0.052). In the test phase a significant discrimination was observed when no perceptual cue was controlled for (t(8) = 2.449, p = 0.020) and when the total luminance was controlled for (t(8) = 2.310, p = 0.025); no significant choice toward the trained quantity was found when the sum of perimeter of the figures (t(8) = 1.316, p = 0.225), the cumulative surface area (t(8) = −1.512, p = 0.169), and the overall space occupied by the arrays (t(8) = −0.373, p = 0.719) were controlled for (Fig. 3a).

However, since area and perimeter of the figures are strictly related to each other, in this experiment by controlling one variable we inevitably affected the other, so that it was not possible to conclude whether one or both variables were important in the discrimination.

### Experiment 1.b. Area vs. perimeter

We accordingly set up another experiment with ten subjects, using the same procedure as before in pre-training and training phases, whereas in the test phase fish were presented with only two different sets of stimuli: one set in which the cumulative surface area was paired whereas the sum of perimeter was not (i.e. the perimeter could suggest the exact ratio between the quantities), and one set in which the sum of the perimeter was paired whereas the cumulative surface area was not (i.e. the area could suggest the exact ratio between the quantities).

We reported no difference in the proportion of correct choices between fish trained with three (mean±std. dev.: 0.788±0.066) and those trained with two figures (0.753±0.065; t(8) = 0.832, p = 0.429). When the relative ratio of the areas (but not the perimeter) was predictive of the numerical ratio, we observed a significant choice toward the trained quantity (t(9) = 3.786, p = 0.004) whereas no significant choice was observed in the condition in which the perimeter, but not the area, could be used to distinguish between two quantities (t(9) = −0.653, p = 0.530). The difference between the two conditions was significant (paired t-test, t(9) = 2.865, p = 0.019, Fig. 3b).

On the whole, results of Experiment 1 showed that fish were found to base their discrimination on the cumulative surface area occupied by figures and on the overall space occupied by the arrays, while they did not use the sum of perimeter, the total luminance of the stimuli or the number of items. Interestingly, in Experiment 1.a we observed a negative correlation between the proportion of correct choices when cumulative surface area was paired and when the overall space of the arrays was paired (Spearman test, ρs = −0.734, p = 0.024) indicating that there was an individual variability in the cues used with some subjects relying on the cumulative surface area for discriminating and not being affected by the overall space, while others used the overall space but not area to solve the task.

### Experiment 2. Discrimination of small quantities using only numerical information

In this experiment we trained fourteen fish to discriminate between 2 and 3 objects while we simultaneously controlled stimuli for their non-numerical variables in both the pre-training and the training phase, with the aim of determining whether fish could learn the discrimination using only numerical information. Using the same geometric figures as the previous experiment, we designed pairs of stimuli in which the total luminance, the cumulative surface area, and the overall space occupied by the arrays were paired between the groups with two and three elements. We found no difference in the proportion of correct choices between fish trained with three (mean±std. dev.: 0.690±0.037) and those trained with two figures (0.651±0.070; t(12) = 1.328, p = 0.209). All 14 fish reached the criterion (chi square test, p<0.05), proving thus able to select the trained numerosity. Overall the choice for the trained stimuli is highly significant (t(13) = 11.103, p<0.001).

As a by-product of controlling for three perceptual variables, stimuli differed for two other non-numerical variables that the fish could have used instead of number to learn the discrimination. The by-product of pairing the cumulative surface area between sets with two and three elements was that in the latter sets smaller-than-average in area figures were more frequent. The by-product of pairing the overall space occupied by configuration was that figures were more spaced out in the sets containing two figures. After reaching criterion, fish were thereby subjected to a test phase without reinforcement using pairs of stimuli composed of figures of identical size and similarly spaced.

Results showed that fish still significantly selected the trained numerosity, even when all the elements were equal to each other and the density of the elements was controlled for (t(13) = 4.397, p = 0.001).

When we compared the number of trials necessary to reach criterion in Experiment 1 (when all numerical and non-numerical cues were available) and Experiment 2 (where only numerical cues were available), we found no difference between experiments (trials in Experiment 1: 25.2±11.7; trials in Experiment 2: 29.14±9.7; F(1,33) = 1.064, p = 0.170; power = 0.170).

## Discussion

Our experiments show that the ability of mosquitofish to discriminate among sets containing a different number of elements is not limited to the socio-sexual context [15], [22], [23], but also applies to sets of abstract elements. They also indicate that mosquitofish can accomplish this task when all non-numerical perceptual variables are matched between the stimuli, thus strongly suggesting that teleosts, like mammals, possess true counting abilities, at least in the domain of small numbers.

### Cues spontaneously used by fish to discriminate between quantities

The first experiment showed that during the extinction phase a good performance was maintained when no control of perceptual variables was operated or after the total luminance of the stimuli and the sum of perimeter of figures were matched. Conversely mosquitofish were unable to select the learned numerosity when stimuli were matched for the cumulative surface area or for the overall space occupied by the arrays, thus suggesting that these two cues had been used during the learning process. Previous works have already demonstrated that such variables play an important role as proxies of numerosity in humans and other mammals [12], [18], [19]. It is interesting to notice that the finding of Experiment 1.a, that fish are influenced by two different non-numerical cues, is somehow a paradox. In fact, because this experiment only controlled for one variable at a time, the fish should have succeeded in all conditions. That is, when the cumulative surface area was controlled for, the overall space of the arrays was not, and vice versa. One explanation for this apparent conflict is that fish combined two different non-numerical cues to learn the numerical discrimination, and that, consequently, the absence of one of the two cues was sufficient to worsen their performance. A second possibility is that fish used only one non-numerical cue, but that there was individual variation in the cue they adopted to learn the discrimination. Our data are more in accordance with the latter hypothesis. In Experiment 1.a the performance of subjects in trials when the cumulative surface area was controlled for correlated negatively with the performance in trials when the overall space occupied by the arrays was controlled for, suggesting that fish that were more influenced by the cumulative surface area were unaffected by manipulation of the overall space of the arrays, whereas subjects that relied on this latter cue did not use the cumulative surface area during the learning process. However, this evidence is based on the examination of a small number of subjects, and caution should be exercised before drawing firm conclusions on this question.

### Discrimination of small quantities using only numerical information

Results of the second experiment showed that fish can use numbers when perceptual variables that correlate with numerosity were excluded. To date, this is the first evidence that a lower vertebrate can really represent and compare numbers. The capacity to discriminate among sets containing different numbers of objects by using numerical information only, previously reported for six month old infants [24], primates, dolphins and dogs [4], [10], [11], [25], is here extended to include a species, the Eastern mosquitofish, which is phylogenetically very distant from mammals and has a much smaller brain size compared with the former species.

Observations made in this study were limited to a single quantity discrimination, 2 vs 3. This was shown to be the upper limit in the capacity of discrimination of six-month old infants [18]. In experiments with continuous variables controlled for, non-human primates successfully discriminate between 3 and 4 objects [4], and non-verbal counting abilities of human adults can be even better [26]. Mosquitofish have been shown to discriminate a shoal of three fish from one of four in two different contexts [15], [22], but in these experiments access to continuous extent of the stimuli could not be prevented. In one of these studies [22], shoals were in two distinct compartments of the apparatus so that they could not be seen and compared simultaneously. This implies that whatever information, numerical or continuous, the mosquitofish encoded, they were able to maintain it temporarily in working memory. Further experiments are necessary to determine whether the upper limit of discrimination of fish also matches that of mammals when access to continuous extent of the stimuli is prevented.

Many authors now agree that there are two distinct non-verbal systems for representing numerosity in animals, adults and human infants [7], [17]. The first mechanism proposed is the one most likely investigated by us in this study. It is an object-tracking system that operates on a small number of items by keeping track of individual objects [27], [28]. It is precise but, due to the limited number of available indexes, it is supposed to allow for the parallel representation of up to 3–4 elements only [29]. The second is an analog magnitude system of numerical representations that allows approximate discrimination of large quantities. It obeys Weber's Law, which holds that as numerical magnitude increases, a larger disparity is needed to obtain the same level of discrimination [8], [30]. Fish have shown to rival primates in their ability to discriminate large quantities approximately [15], [22], [31], [32]. However, while controlled experiments have shown that six month babies and non-human primates can perform large number discrimination using only numerical information, no such evidence exists for fish. Future research should assess if the analog magnitude system can operate in fish when access to continuous extent of the stimuli is prevented.

### Number as a last resort?

Comparison of the two experiments suggests that although mosquitofish are capable of using both number and continuous extent, they spontaneously use the latter to estimate quantities. Similar results have been reported to occur in dolphins, macaques, six month old infants and human adults [10], [16], [18], [19], [27]. For instance, a bottlenose dolphin trained to distinguish between two quantities spontaneously used overall surface area of the elements or brightness for performing the discrimination [10]. However, controlling for non-numerical cues, these authors demonstrated that the dolphin could discriminate the stimuli solely on the basis of the numerosity feature and that eventually it was able to successfully transfer the discrimination to novel numerosities outside the former range.

Traditionally, the explanation for these results is that number requires more effortful processing compared with continuous extent, and therefore counting represents a ‘last resort’ strategy, when no other perceptual mechanism may suggest the quantity of the elements [10], [18], [33]–[35]. However, recent studies have questioned this assumption, showing that adult humans, pre-verbal children, chimpanzees and macaques spontaneously and automatically encode information about continuous extent and numerosity simultaneously, and that the relative salience of these two dimensions depends on factors such as type of task, numerosity ratio and previous experience [17], [36]–[39].

Recently, Burr and Ross [40] have provided evidence for a putative physiological mechanism underlying this capacity. After being exposed for 30 sec to a large number of spots in one portion of their visual field, the subjects of this experiment tended to underestimate by three times the number of spots being subsequently presented in the same region of retina. The presence of a retinotopic adaptation clearly indicates that the visual system is able to extract, at an early stage, the numerical information from a visual scene, just as it extracts other ‘primary visual properties’ such as colour, size, orientation and spatial frequency.

Our study was not designed to specifically investigate this issue. However we found that learning was equally effective in the first experiment when subjects could use all physical properties of the stimuli and in the second, when they had access only to the numerical cues, suggesting that the precedence of perceptual cues is not determined, at least in fish, by a greater cognitive effort when numerical computation is involved.

Why do mosquitofish preferentially use continuous extent over numerical information given that the two alternatives are similar in cognitive demand? One possibility is that quantity information is ecologically more relevant for this species. For example, in foraging contexts animals often tend to maximise the amount of resources acquired with a minimum of energy expenditure [41], [42]. Even though number of items and total amount of resource gained frequently correlate, sometimes this does not occur, for example when there is a large variation in the size of food items. Selection for optimising food intake could have favoured mechanisms based on continuous extent, such as area, as they are more reliable indicators of the resource potentially gained [43], [44]. Alternatively, perceptual cues of the stimuli may simply be the quickest indicator of the numerosity, for example because they involve earlier stages in neural visual or auditory processing. Mosquitofish use quantity discrimination in fitness related contexts, such as choosing the safer social group or the larger number of potential mates [15], [23], in which speed of decision is often crucial. Mechanisms based on continuous extent may have been favoured in this species since they allow choosing the best option in the fastest way. One recent study with adult humans [38] has provided evidence that the extraction of a representation of continuous extent, such as the area of stimuli, in most cases proceeds more rapidly than the extraction of a representation of discrete quantity. There is some evidence that this may be the case for rats and pigeons too [34], [45], suggesting that it may represent a common property of vertebrate visual system.

In summary, this study provides a new insight into the evolution of cognitive abilities of vertebrates. Many authors have proposed the existence of shared mechanisms for non-verbal numerical discrimination in humans, non-human mammals, and birds [4], [14], [27], [46]. The present results provide further evidence that is coherent with previous works [15], [22], [31], [47], indicating a fundamental similarity of mechanisms underlying non-verbal numerical abilities in distantly related vertebrates and reinforcing the idea that numerical systems may be more ancient than we had previously assumed.

## Materials and Methods

### Experiment 1.a. Cues spontaneously used by fish to discriminate between quantities

#### Subjects

Ten female Eastern mosquitofish (*Gambusia holbrooki*) were used as subjects of this experiment. Fish were collected from Valle Averto, a system of brackish water ponds and ditches in the Venetian lagoon basin (northern Italy), returned to the laboratory and initially maintained in small mixed-sex groups (12–15 fish, approx. 1∶1 sex ratio) kept in 70-l glass aquaria with abundant vegetation (*Vesicularia dubyana* and *Ceratophyllum demersum*), lit by a 15 W fluorescent lamp (16L∶8D) and with a water temperature that was maintained at 25±2°C. Subjects were used once; companion females, on the other hand, were used more than once.

#### Apparatus and Stimuli

**Figure 4:**
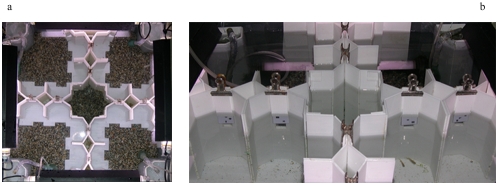
Pre-training apparatus. Aerial (a), and lateral view (b). Eight pairs of equal doors allowed fish to move between the four compartments. Stimuli (3 figures or 2) were placed above each door and only the door below the reinforced quantity permitted the passage.

*Pre-training phase.* One week before the training, fish were placed in a 68×68×38 cm tank, divided into four equal sectors by white plastic partitions (Fig. 4). The tank was lit by four fluorescent lamps positioned around the borders, and water was maintained at a temperature of 25°±2°C. The bottom was covered with natural gravel, and vegetation (*Vesicularia dubyana*) was provided as well as aquarium filters.

To move between sectors, each partition was provided with two doors of equal size (2.5×3.5×1 cm) closed by a flexible plastic material and located 12.5 cm from the floor of the tank, with a distance of 8 cm between them. Above each of the two doors we placed two identical stimuli, each occupying a 3×3 cm area. Each stimulus set contained one exemplar with two elements and one with three. Elements were geometric figures differing in shape, size and luminance, randomly chosen from a set of approximately 100 elements and positioned on a white background. The average distance among elements in stimuli containing two or three elements was the same (see examples in Fig. 2).

Only the door below the reinforced quantity permitted them to pass from one sector to the other. This was achieved by gluing the transparent material on the top of the door, so that fish could easily bend it and pass through the door. On the other door the transparent material was glued also at the bottom, so that fish could not pass through. An openable door could be traversed in both directions, and pairs of stimuli were placed on both sides of the partition so that a total of 8 different pairs were presented inside the tank at the same time. These stimuli were changed daily, and a total of 56 different pairs of stimuli were used during the pre-training phase.

The experimental apparatus (Fig. 1) was used in the training phase and in the test phase. It consisted of a small white test chamber (16×16×16 cm) inserted in a larger tank (60×26×36 cm) to provide a comfortable area with vegetation and food where the test fish was placed together with other three companion females, 10 minutes before starting the training session. The tank was inserted in a dark room and covered with a one-way screen to eliminate extra-tank cues. Female mosquitofish are highly social and spontaneously tend to join the other females when placed in an uncomfortable environment [15]. Previous work has shown that this procedure provides motivation for social reinstatement in fish [48].

At two corners of the chamber, two small tunnels (3×4×2.5 cm, located 2 cm from the floor of the tank) made from white plastic material were inserted, allowing the fish to pass through it to rejoin conspecifics in the outer tank. At the end of each tunnel there was a door similar to that used in the pre-training tank. As previously, one door was blocked, while the other could be opened by bending the flexible plastic material.

Sixty new pairs of stimuli were used, with the same characteristics of those used in the pre-training phase. As the elements of the stimuli were randomly selected, during pre-training and the training phase fish could learn to distinguish between two quantities by using both number and non-numerical information that correlated with number, such as the cumulative surface area or the overall space occupied by the arrays. Conversely, in the control test, five different sets of stimuli were presented. In four, we controlled for one continuous variable at time, namely the cumulative surface area of the elements, the total luminance of the stimuli, the sum of perimeter, and the overall space occupied by the arrays. The fifth was a control set of stimuli, in which no control for non-numerical variables was performed. All stimuli were created by using Microsoft Office 2003 and the area, perimeter and luminance was controlled using TpsDig software.

#### Procedure

Three different steps were planned: the pre-training, the following training phase and the test phase. Half of the subjects were trained toward the larger quantity (three), whereas the second half were trained toward the smaller one (two). In the first step, two subjects were kept for 7 days inside the pre-training tank. All the couples of stimuli were changed daily and fish were left free to swim inside the four sectors without any interference from the experimenter for the whole period.

At the beginning of day 8, all fish commenced the training phase in the experimental apparatus: fish were singly tested each day (6 trials per day) from a minimum of three to a maximum of ten days. During the trials, fish were brought to the test tank by inserting them into a transparent plastic cylinder (4.5 cm in diameter) and placing it in the centre of the test chamber. After 30 seconds, the cylinder was removed, leaving the fish in the middle of the test chamber. The first door they initially reached was recorded until the fish was able to exit and rejoin conspecifics (the maximum time allowed to exit was 20 minutes). Inter-trial intervals lasted 5 minutes, during which the fish was allowed to shoal with the conspecifics; in the meantime the experimenter changed the pair of stimuli. The location of the trained quantity was exchanged at any successive trial. Furthermore, since the subject was disoriented between successive trials and no external cue was available, the two corners were equivalent from the point of view of the fish, reducing any possibility that fish may have preferentially chosen one door on the basis of the geometrical information of the environment [48].

The learning criterion was a statistically significant frequency of correct choice estimated with chi square test. Starting from day 3, we statistically analysed the daily performance of the subject, and once discrimination reached significance it was admitted the next day to the following test phase. Procedure for this phase was similar to that used during the training phase, with the exception that we adopted an extinction procedure by keeping both doors blocked. The first choice was recorded until a maximum period of 2 minutes. After this period, fish were released outside the test tank and could join their conspecifics; 5 minutes later, the subject was re-inserted into the test chamber in the presence of a new pair of stimuli. This phase lasted 5 days, with 6 trials per day, for a total of 30 overall trials, 6 for each set of stimuli. The five sets were randomly intermingled during each daily session. Statistical tests were conducted using SPSS 15.0.

### Experiment 1.b. Area vs. perimeter

Ten female mosquitofish were used as subjects. Experimental apparatus and procedure were the same described in Experiment 1.a. The same stimuli described in Experiment 1.a were used in pre-training and training for this experiment. For the test phase, fish were presented with only two sets of stimuli. In one, the cumulative surface area of the stimuli was exactly paired, whereas the relative ratio of the perimeter between the groups - 3 and 2 elements - was equal to 3/2 (the perimeter could then suggest the exact ratio between the quantity whereas the area could not); in the other set we used an opposite pattern, controlling for the perimeter but having the area that could suggest the exact ratio between the quantities (3/2). In both cases we paired stimuli for the overall space occupied by the arrays. During each daily session, half of the trials presented the former set whereas the remaining presented the latter set. The two sets were randomly intermingled within each session.

### Experiment 2. Discrimination of small quantities using only numerical information

#### Subjects and apparatus

A total of 14 female mosquitofish were used as subjects. Apparatus was the same as for the previous experiment.

#### Stimuli and Procedure

The procedure for this experiment was similar to the previous one, with the exception that during pre-training and training phases we used pairs of stimuli in which the cumulative surface area, the total luminance and the overall space occupied by the arrays were simultaneously controlled for. The key phase for this experiment was the training phase, since we aimed at determining whether fish could learn the discrimination in the absence of non-numerical cues. During the training phases of this experiment all subjects received the same number of trials, 36, comprising 6 trials per day for a total of 6 days. As before, the criterion for discrimination was a statistically significant frequency of correct choices during the training phase.

By pairing the cumulative surface area and the overall space occupied by the arrays we could have provided subjects with two additional non-numerical cues. In each pair, the stimulus with the larger number of elements (three) tended, inevitably, to contain small elements more often than the corresponding stimulus with two elements. By occupying the same overall space, the stimulus with three elements also tended to have a shorter distance between the elements. Both cues could, in principle, be used by fish to learn the discrimination. We therefore added a test phase, in which we presented with an extinction procedure pairs of stimuli in which all elements were identical in size and shape (all circles, all stars, etc.) and were similarly spaced. Fish received a total of 24 trials (6 trials per day, for 4 days).
